# MAKING AN IMPACT: AN INTEGRATED MODEL TO REDUCE AGEISM AND SUPPORT PROFESSIONAL IDENTITIES

**DOI:** 10.1093/geroni/igad104.2117

**Published:** 2023-12-21

**Authors:** Amy Powell-Versteeg, Rebekah Perkins, R Scott Ward, Linda Edelman, Katherine Supiano

**Affiliations:** University of Utah, Salt Lake City, Utah, United States; University of Utah, Salt Lake City, Utah, United States; University of Utah, Salt Lake City, Utah, United States; University of Utah, Salt Lake City, Utah, United States; University of Utah, Salt Lake City, Utah, United States

## Abstract

Nearly one-million people in the U.S. are living with Parkinson’s disease (PD) and require skilled care. Despite this, only 20% of educational programs offer a geriatric rotation. Ageism is a major factor in healthcare students’ lack of clinical exposure, decreased self-confidence, and desire to specialize in geriatrics. Research shows positive exposure to older adults improves students’ knowledge, attitudes, and interest in this population. Yet, limited geriatric curriculum exists that integrates specialized knowledge combined with positive contact experiences to shape students’ professional identities as capable providers. This project combined two experience-based learning (ExBL) models to form the Intrinsically-Motivated learning, Professional identity, Awareness, Capacity, and Timing (IMPACT) model—to support specialized knowledge acquisition and positive, repeated contact experiences with older adults. Third-year Doctor of Physical Therapy (DPT) students who completed both didactic training and a 2-week PD Boot Camp were interviewed about their experiences in the program. Nine students participated in the PD Boot Camp and interviews. Qualitative thematic analysis revealed the following: 1) unexpected insights gained about clients’ unique abilities, 2) therapeutic alliances formed and mutually created functional goals, 3) improved decision-making through practice with mentorship, and 4) increased self-confidence to provide care to clients with complex conditions. The IMPACT model provided students specialized knowledge and practical skills to capably care for older adults with PD. This model can serve as a guiding educational framework to reduce ageism among healthcare students across a variety of settings, support students in developing their professional identities, and increase interest in caring for older adults.

